# **Erratum notice for:** “Suppressive effect of platycodin D on bladder cancer through microRNA-129-5p-mediated PABPC1/PI3K/AKT axis inactivation” [Braz J Med Biol Res 2021;54(3):e10222]

**DOI:** 10.1590/1414-431X2022e10222erratum

**Published:** 2022-06-22

Dayin Chen^1,2^ https://orcid.org/0000-0002-6810-0521, Tingyu Chen^3^ https://orcid.org/0000-0001-6339-5503, Yingxue Guo^1^ https://orcid.org/0000-0003-1238-6767, Chennan Wang^1^ https://orcid.org/0000-0001-6846-1805, Longxin Dong^1^ https://orcid.org/0000-0002-6383-6476, and Chunfeng Lu^1,3^ https://orcid.org/0000-0002-1978-3266

^1^Department of Pharmacology, Basic Medical College, Jiamusi University, Jiamusi, Heilongjiang, China

^2^Department of Urology, the First Affiliated Hospital of Jiamusi University, Jiamusi, Heilongjiang, China

^3^School of Medicine, Huzhou University, Huzhou, Zhejiang, China

Correspondence: Chunfeng Lu: <chunfenglu883@163.com>

Erratum for: Braz J Med Biol Res | doi: 10.1590/1414-431X202010222

The authors notified the Editors of the Brazilian Journal of Medical and Biological Research that ‘Panel D of Figure 3 did not show good results. It should be replaced with the experimental results that are most consistent in the three replicates’. They guarantee that this modification of Figure 3D does not change the findings of their research.

The correct Figure 3 is:

**Figure 3:**
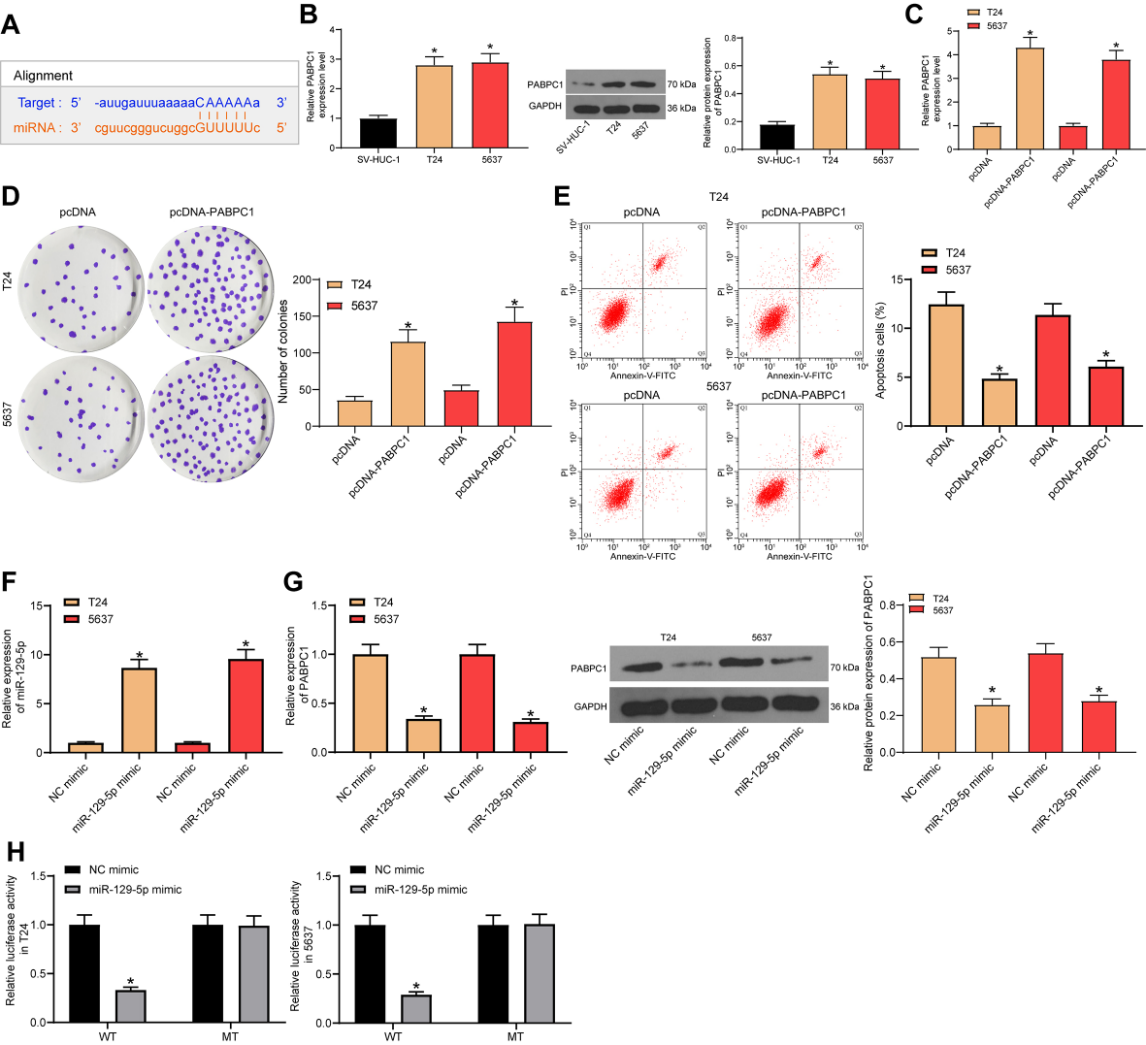
miR-129-5p directly targets PABPC1. **A**, Targeting between miR-129-5p and PABPC1 predicted using StarBase (http://starbase.sysu.edu.cn/). **B**, mRNA and protein expression of PABPC1 in bladder cell lines (T24 and 5637) and a human uroepithelial cell line (SV-HUC-1) were determined by RT-qPCR and western blot analysis, respectively (*P<0.05 compared to SV-HUC-1 cells, one-way ANOVA). **C**, PABPC1 expression in bladder cancer cells after pcDNA-PABPC1 transfection was determined by RT-qPCR. **D**, Colony formation ability of cells was determined by colony formation assay. **E**, Apoptosis rate of bladder cancer cells was determined by flow cytometry. **F**, miR-129-5p expression after miR-129-5p mimic transfection was determined by RT-qPCR. **G**, mRNA and protein expressions of PABPC1 in cancer cells after miR-129-5p mimic transfection were determined by RT-qPCR and western blot analysis, respectively. **H**, Relative luciferase activity in cells after co-transfection of PABPC1-WT/MT vector and miR-129-5p mimic/mimic control. Data are reported as means±SD. *P<0.05 (one-way ANOVA). Three independent experiments were performed.

